# Molecular basis for pH sensing in the KDEL trafficking receptor

**DOI:** 10.1016/j.str.2024.03.013

**Published:** 2024-04-15

**Authors:** Zhiyi Wu, Kathryn Smith, Andreas Gerondopoulos, Tomoaki Sobajima, Joanne L. Parker, Francis A. Barr, Simon Newstead, Philip C. Biggin

**Affiliations:** 1 Structural Bioinformatics and Computational Biochemistry, Department of Biochemistry, University of Oxford, Oxford OX1 3QU, UK; 2 Department of Biochemistry, University of Oxford, Oxford OX1 3QU, UK; 3 Kavli Institute for Nanoscience Discovery, University of Oxford, Oxford OX1 3QU, UK

## Abstract

Trafficking receptors control protein localization through the recognition of specific signal sequences that specify unique cellular locations. Differences in luminal pH are important for the vectorial trafficking of cargo receptors. The KDEL receptor is responsible for maintaining the integrity of the ER by retrieving luminally localized folding chaperones in a pH-dependent mechanism. Structural studies have revealed the end states of KDEL receptor activation and the mechanism of selective cargo binding. However, precisely how the KDEL receptor responds to changes in luminal pH remains unclear. To explain the mechanism of pH sensing, we combine analysis of X-ray crystal structures of the KDEL receptor at neutral and acidic pH with advanced computational methods and cell-based assays. We show a critical role for ordered water molecules that allows us to infer a direct connection between protonation in different cellular compartments and the consequent changes in the affinity of the receptor for cargo.

## Graphical abstract

**Graphical abstract:**
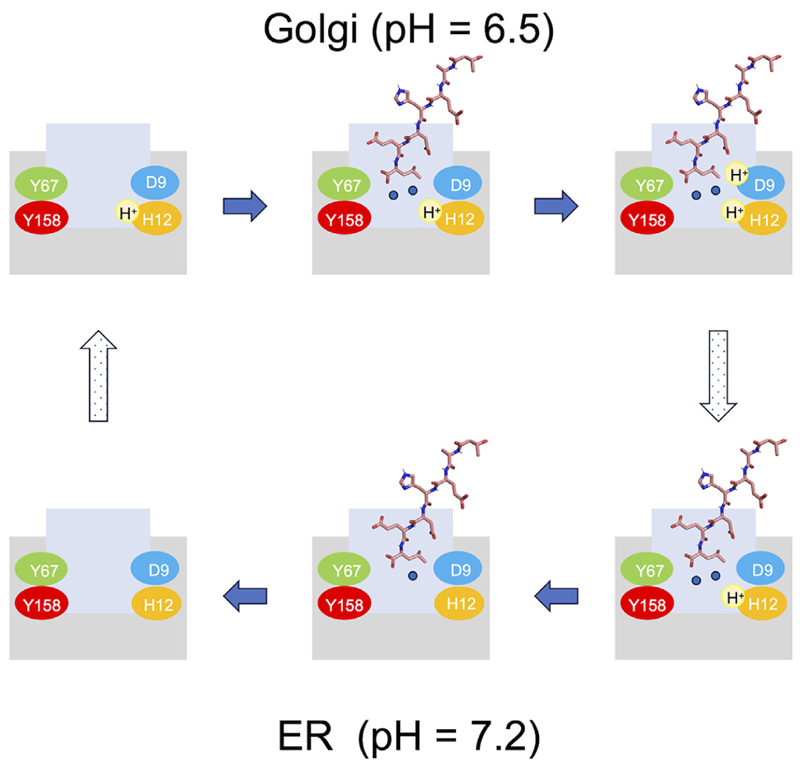

## Introduction

Maintaining the integrity of organelles is a fundamental property of eukaryotic cells and is controlled through tightly regulated protein trafficking pathways.^1^ Many proteins are targeted to specific organelles through the presence of signal peptides, which interact with distinct trafficking receptors and which, in turn, control their localization through packaging into coated vesicles.^2^ An important characteristic of organelle identity is luminal pH. Specific proton concentrations result from the activation of the v-type ATPase proton pump in combination with different ion channels, transporters, and lipids in the different organelles.^3^ Despite this understanding, the molecular mechanisms by which changes in luminal pH are sensed and linked to organelle maturation and protein trafficking are less well understood. Within the early secretory pathway, newly synthesized proteins in the endoplasmic reticulum (ER) are packaged into coat protein complex II (COPII)-coated vesicles and transported to the Golgi apparatus, where they undergo post-translational modification and quality control.^4^ As part of the folding and trafficking cycle for newly synthesized proteins, luminal ER chaperones and quality control enzymes must be retrieved from the Golgi and returned to the ER.^4,5^ The selective capture and retrieval of ER luminal proteins is controlled by the KDEL receptor (KDELR), which recognizes a specific ER-retrieval signal sequence (ERS) Lys-Asp-Glu-Leu (KDEL) at the C terminus of cargo proteins.^6–8^ Variants of the canonical KDEL retrieval sequence exist in mammalian cells, which vary in their affinity for the receptor.^9,10^ The different sequences, which predominantly vary in the –4 position (numbered from the C-terminal carboxyl group), enable the receptor to respond dynamically to varying concentrations of cargo proteins. Low-abundance chaperones contain the higher affinity HDEL sequence, facilitating efficient retrieval against the higher abundance but lower affinity KDEL-containing proteins.^9,10^ The relationship between the different variations of the KDEL ERS enables the receptor to maintain a high dynamic range yet remain at sub-stoichiometric concentrations relative to its cargo.^11^

At steady state, the KDELR is mainly localized to the early or *cis*-Golgi, where it can efficiently capture ER luminal cargo.^12,13^ The binding of a cargo protein bearing a C-terminal KDEL sequence to the receptor triggers the incorporation of the receptor-cargo complex into COPI vesicles.^14,15^ COPI vesicles return the complex to the ER, where the cargo dissociates, and the receptor is rapidly trafficked back to the Golgi apparatus via the COPII complex system.^16^ Changes in luminal pH between the ER and Golgi play a significant role in regulating the function of the KDELR.^10^ In the mildly acidic environment of the Golgi, KDEL-containing ER luminal proteins bind the receptor with high affinity, which elicits a signal for the integration of receptor-cargo complexes into COPI vesicles.^7^ In contrast, the neutral pH of the ER results in cargo release and the subsequent relocation of the empty receptor back to the Golgi, resetting the retrieval cycle.^4,17^ The KDELR, therefore, links protein localization to organellular pH changes, which is a hallmark of organelle identity. However, the mechanism through which the KDELR senses changes in the luminal pH environment remains unresolved.

Crystal structures of the chicken KDELR revealed a seven transmembrane (TM) spanning integral membrane protein with structural homology to the PQ-loop superfamily of membrane transporters.^11,18,19^ Available structures capture the receptor in both the apo (pH 9.0) and peptide-bound states (pH 6.0), revealing that peptide recognition on the luminal side of the membrane occurs in a large polar cavity that extends from the luminal side of the membrane into the center of the receptor. The structures revealed conformational changes that occur upon ERS peptide binding, which results in the movement of TM7 and the presentation of a conserved di-lysine retrieval motif that binds COPI.^20^ Driving the structural transition is the movement of a conserved arginine on TM6, which moves into the binding site to engage the carboxy-terminus of the ERS peptide,^19^ resulting in the subsequent movement of TM7 and presentation of the COPI binding motif. Following retrieval to the ER, the KDELR undergoes deprotonation and releases the ERS and associated cargo protein, enabling TM6 and TM7 to adopt the resting conformation, which signals to COPII to return the receptor to the Golgi via exposure of an acidic motif.^19,21^

A key unresolved question is how the receptor senses the change in luminal pH following trafficking from the Golgi back to the ER. *In vitro* binding and cellular retrieval assays highlighted an essential role for a conserved histidine (His12) in peptide binding.^19,21^ Due to the protonatable nature of this residue within the physiological range of the secretory pathway, it was proposed that this side chain may form the pH sensor for the KDEL retrieval system.^19^ The subsequent crystal structure of the KDELR2 protein revealed that His12 is located adjacent to a short hydrogen bond (SHB) formed between conserved tyrosine (Tyr158) and glutamate (Glu127), which functions to lock the receptor in an active state following binding of the KDEL signal peptide.^19^ Using molecular dynamics, we previously demonstrated that protonation of His12 stabilizes this SHB;^22^ however, the mechanism linking His12 protonation and ERS binding remained unclear due to the lack of additional structural information on the deprotonated state of the system.

Therefore, to understand how the KDELR senses luminal pH in the ER, we determined the crystal structure of the chicken KDELR2 protein in complex with TAEHDEL peptide under different pH conditions. Combined with our previous KDELR structures, our results confirm that His12 indeed functions as the pH sensor in this system. Further computational methods reveal that deprotonation of His12 likely disrupts an ordered water network within the binding site, destabilizing the receptor’s ERS-bound state, thus explaining rapid cargo release in the ER. Our results now provide a detailed mechanism for proton movement between His12 and the ER lumen, explaining the relationship between the ERS and the essential role played by water in enabling the receptor to sense and respond to pH changes within the early secretory pathway.

## Results

### Structure of KDELR bound to TAEHDEL peptide at pH 7.0

**Figure 1:**
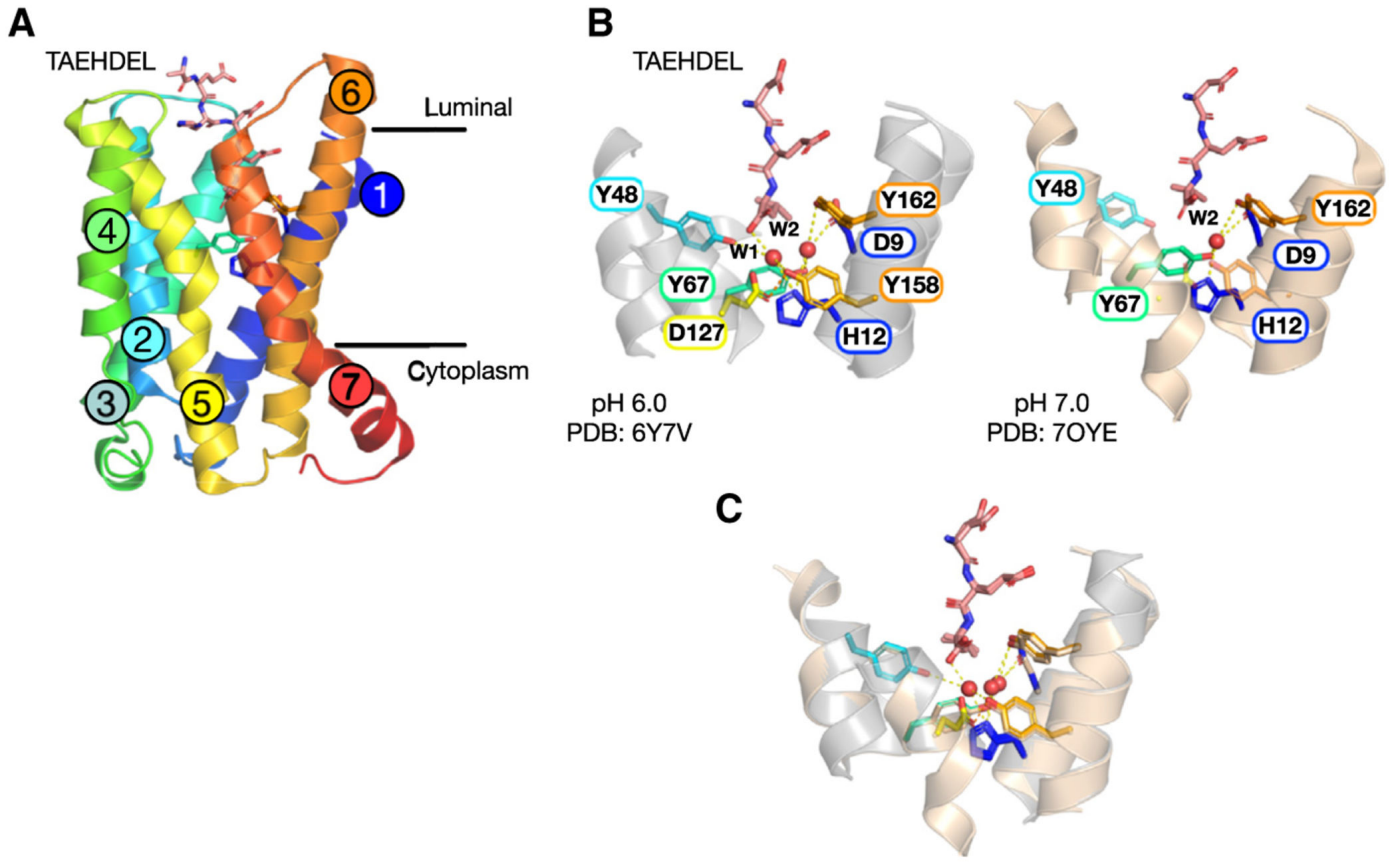
Structures of the KDEL receptor (A) Cartoon depicting the overall fold of the receptor (colored from N terminus blue to C terminus; red) when in complex with the TAEHDEL peptide ligand (pink). (B) Close-up views of the binding site at pH 6.0 (left and gray helices) and 7.0 (right and wheat helices) showing the change in the water network at the base of the binding pocket. (C) Overlay of the structures at pH 6.0 and 7.0.

We first sought to obtain structural insights into the transient ER form of the KDELR-cargo complex, which exists following delivery from the acidic environment of the Golgi. To capture a ligand-bound structure of the chicken KDELR at neutral pH, we took advantage of the higher affinity observed for the TAEHDEL peptide compared to the TAEKDEL variant, K_D_ 0.24 μM vs. K_D_ 1.94 μM, respectively.^11^ Crystals of the KDELR bound to the TAEHDEL peptide were grown at pH 7.0 using the lipidic cubic phase method and diffracted X-rays to 2.6Å (Table 1). The receptor adopts the same overall conformation observed previously for the TAEHDEL-bound complex crystallized at pH 6.0 (PDB:6y7v),^11^ with a root-mean-square deviation of 0.283 Å over 208 Cα atoms. The receptor adopts the active state, with TM7 kinked out exposing the di-lysine COPI retrieval signal (Figure 1A). Previous structural studies on the KDELR bound to either TAEKDEL or TAEHDEL peptides revealed two ordered water molecules (W1 and W2) at the base of the binding cavity.^11,19^ The water molecules coordinate interactions between the carboxy-termini of the peptide, Tyr158 on TM6 and His12 on TM1 (Figure 1B).^11,19^ The two water molecules are also present in the apo KDELR bound to an inhibitory sybody Syb37,^19^ suggesting they are present in the receptor before the peptide binds. However, in the structure determined at pH 7.0, only one of the two water molecules (W2) is visible in the electron density maps (Figures 1C and S1). For structures at acidic pH, the water molecule at pH 7.0 coordinates interactions between His12, Tyr67 and Tyr162 on TM2 and TM6, respectively, and Asp9 on TM1 and does not interact with the carboxy-terminus of the TAEHDEL peptide. The loss of the W1 water molecule in the pH 7.0 structure thus collapses the hydrogen bond interaction network observed under acidic conditions, with the carboxy-terminus of the TAEHDEL peptide now only interacting with Arg47 and Arg159 (Figure S1).

A key mechanistic step in stabilizing the peptide-bound state of the receptor is the formation of a short hydrogen bond between Glu127 and Tyr158 on TM5 and TM6, respectively.^19,22^ The formation of this high-energy bond locks Arg159 in a position that captures the C terminus of the signal peptide. We previously showed that His12 protonation stabilizes this SHB, which results in the high affinity of the receptor for the signal peptides.^22^ We reasoned that the loss of the W1 water molecule at pH 7.0 may also be linked to the protonation state of His12, which would mechanistically link the protonation of the receptor to stabilization of the interaction between Glu127 and Tyr158 and the generation of a stable hydrogen bond network observed at pH 6.0. To test this hypothesis, we determined the structure of the His12Ala variant of the KDELR at pH 6.0 bound to Syb37. The structure was determined at 2.3 Å resolution and under identical conditions to the previously reported wild-type (WT)-Syb37 complex (Table 1). At pH 6.0 in the WT protein, the two water molecules are in the same position in both the Syb37-bound complex (PDB:6i6j) and the TAEHDEL complex (PDB:6y7v) (Figure S2A). However, in the His12Ala variant, the electron density maps clearly show the loss of both water molecules in the binding site (Figure S2B), indicating that His12 is both necessary for the stabilization of the SHB and for the stabilization of the water-mediated hydrogen bond network coordinating the carboxy-terminus of the signal peptide. Although histidine is a common side chain in proton transfer reactions in enzymes,^23^ its role in proton-coupled transporters is surprisingly underrepresented.^24^ In contrast, many solute carriers use aspartic acid and salt bridge interactions with lysine and arginine to couple proton binding to conformational changes that drive transport.^25^ Therefore, to understand how the protonation of His12 might influence KDELR function and dynamics, we investigated different protonation states using a series of computational approaches.

### The protonation of His12 occurs at specific sites

Although the crystal structures at neutral and acidic pH conditions revealed a difference in water occupancy within the KDELR, it was not possible to resolve the positions of any protons. To fully understand the mechanism of proton (pH) control, it is necessary to know where the protons are most likely to reside, particularly with respect to His12, which coordinates two the water molecules observed in the crystal structure. To investigate the most likely position of protons on the imidazole ring, we used grand canonical Monte Carlo (GCMC) simulations (see STAR Methods for details) to determine the likely protonation states consistent with the experimental observations.

**Figure 2:**
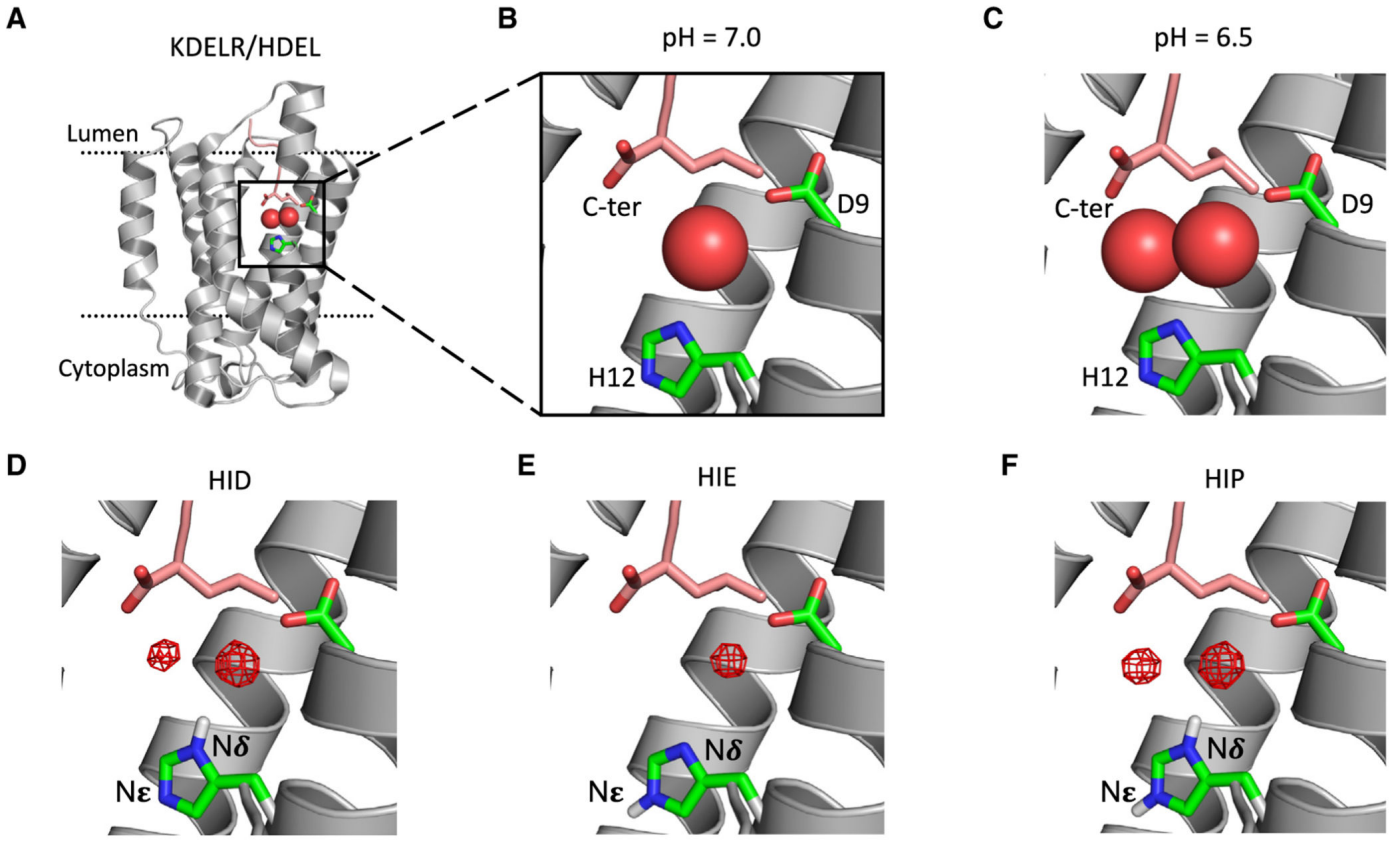
The protonation state of the His12 predicted by GCMC calculations (A) Crystal structure of the KDEL receptor highlighting the position of waters (shown as red spheres) in the binding pocket. (B) Only water is observed in the crystal structure at high pH. (C) Two waters are observed at lower pH. The GCMC calculations show that both HID (D) (protonated at N*δ*) and HIP (F) (both N protonated) could host two waters, while HIE (E) (protonated at N_ϵ_) could only host one water in the binding pocket. The KDEL peptide is shown in pink stick representation.

In the crystal structure at low pH, where two waters are resolved, His12 is most likely protonated to +1 overall charge, with a proton on both the Nδ and Nϵ atoms of the side chain (a state referred to as HIP – see Figure 2). At neutral pH, where only one water was resolved and the His12 has zero overall charge, there are two possible locations for the proton – either on the Nδ (HID) or on the Nϵ nitrogen (HIE). The GCMC calculations show that HIE can only sustain one stable water (Figure 2E), while HID is able to stabilize two waters in the binding pocket (Figure 2D). Considering that the Nϵ of the histidine is pointing toward an enclosed water pocket away from the main ERS binding pocket, the only plausible route for the proton leaving the histidine at low pH will be from the Nδ, which is pointing toward the lumen and has access to the bulk solvent (Figure 2E). Therefore, HIE is the most likely state at pH 7.

### The stability of the water network dictates peptide affinity

**Figure 3:**
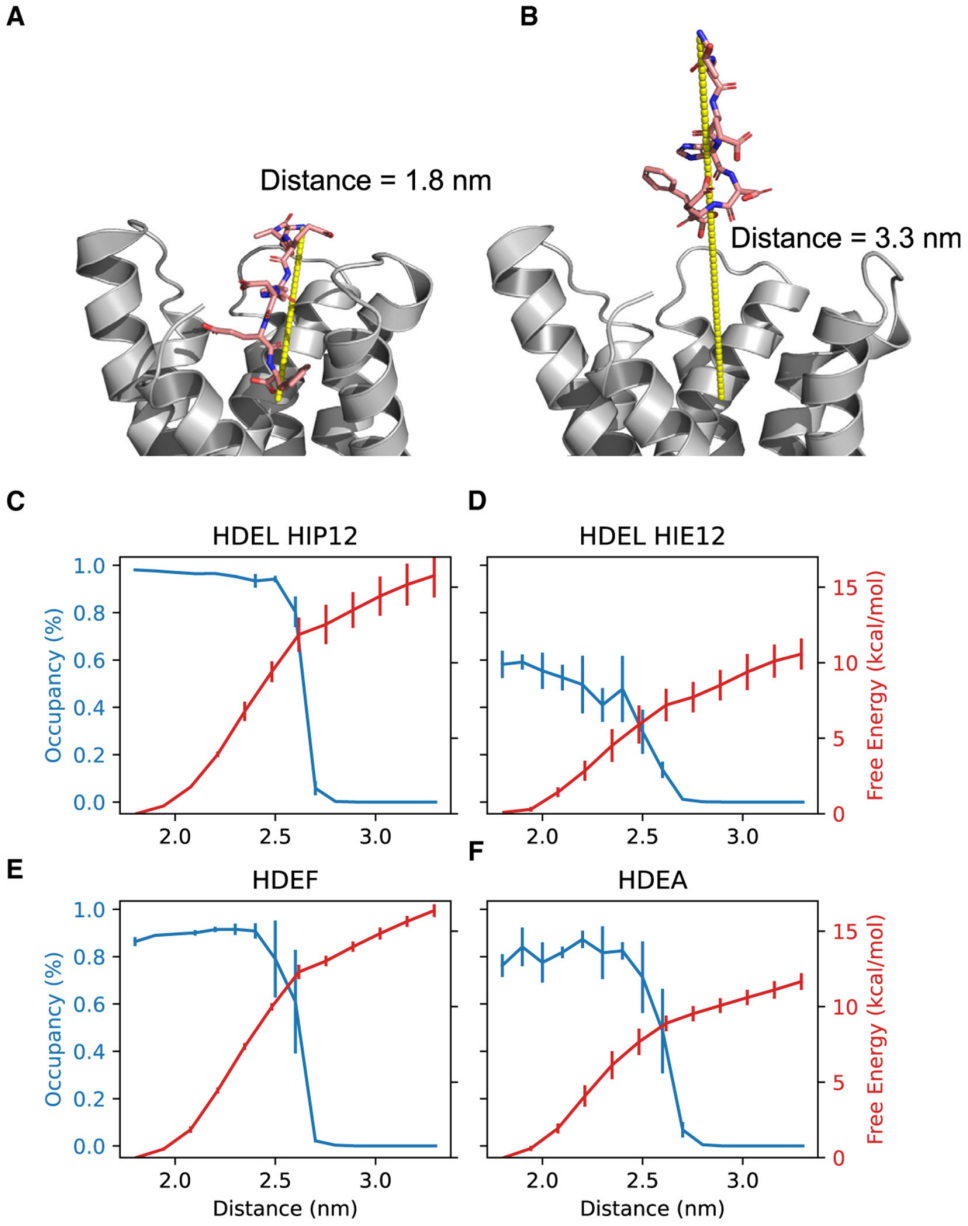
The PMF for ligand unbinding The endpoints are shown in (A) and (B) and reflect the distance between the N-terminal nitrogen of the peptide and center of the binding pocket. For HDEL as a ligand, the unbinding free energy is greater when the H12 is protonated (C: Red) compared with H12 being deprotonated, (D: Red) and the difference is consistent with the increased stability of the two bridging waters (CD: Blue). (E) HDEF can maintain a stable water network, which results in a large unbinding free energy penalty. (F) HDEA, on the other hand, has higher fluctuations and, thus, greater instability (F: blue), resulting in weaker binding. Error bars for (C)–(E) are +/− the standard deviation (n = 3).

Having ascertained the likely protonation state of His12 under the two pH conditions captured in the crystal structures, we next made quantitative assessments of the binding affinity of HDEL-like peptides under different pH conditions. We performed potential of mean force (PMF) calculations to estimate the free energy of removing the HDEL peptide (and other variants, see the following section) from the binding pocket (Figures 3A and 3B). The collective variable was defined as the distance between the binding pocket and the peptide N terminus. When His12 is protonated (HIP12), the free energy that is required to release the HDEL peptide (Figure 3C) is significantly higher than the free energy required when the key histidine is deprotonated (HIE12) (Figure 3D). The decrease in the free energy of peptide unbinding shows that the deprotonation of His12 reduces the binding free energy.

The stability of the water in the binding pocket is defined as the proportion of simulation time that the two waters (W1 and W2) form the water network observed in the binding pocket in the crystal structures (see Figures 1 and 2). When His12 is protonated, then both W1 and W2 remain with 100% occupancy until the C terminus dislodges (Figure 3C). When His12 is deprotonated, the proportion of time in which the two waters are present is only 60%, even in the fully bound state (Figure 3D).

**Figure 4:**
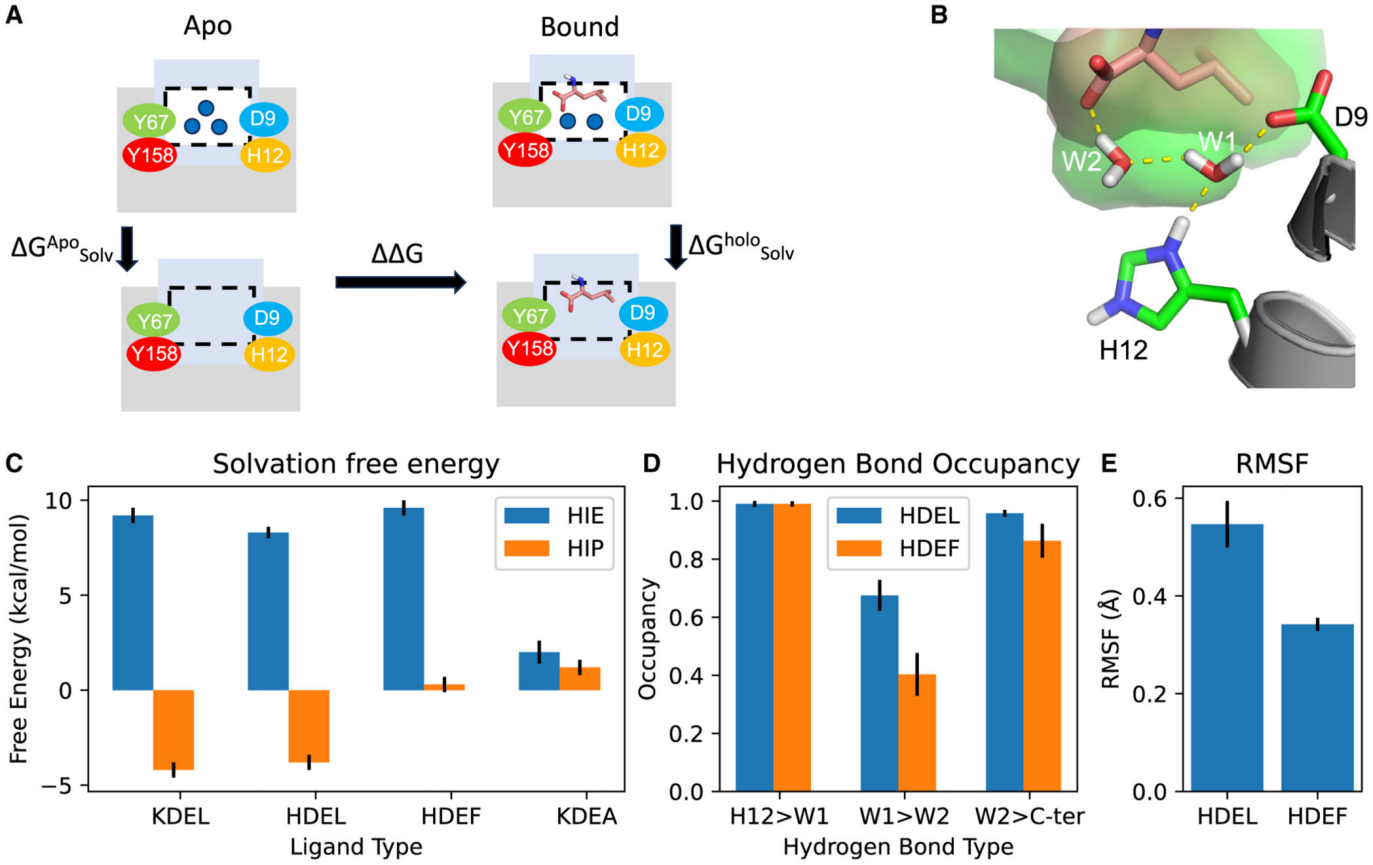
The terminal amino acid modulates recognition of the ERS by the KDEL receptor (A) Endogenous KDEL receptor redistribution was measured in COS-7 cells in the absence (-ligand) or presence of H/KDEL/F/A (mScarlet-xDEx^sec^). TGN46 was used as a Golgi marker. The scale bar is 10 μm. The mean differences for H/KDEL/F/A comparisons against the shared no-ligand control are shown as Cummings estimation plots. The individual data points for the fraction of KDEL receptor fluorescence in the Golgi are plotted on the upper axes. On the lower axes, mean differences are plotted as bootstrap sampling distributions. Each mean difference is depicted as a dot. Each 95% confidence interval is indicated by the ends of the vertical error bars. (B) Cells and media collected from HeLa S3 cells expressing the xDEx variants (mScarlet-K/HDEx^sec^) indicated in the figure were western blotted for resident ER chaperons and KDEL receptor.*unspecific bands (C) K/HDEx secretion to media bar graph showing mean ± SEM (n = 3). (D) Bar graph of endogenous ER chaperones secreted after challenged with different retrieval signals as measured by western blot showing mean ± SEM (n = 3).

To further explore if disturbing the water will disrupt the binding, the terminal leucine at the −1 position in the HDEL ligand was alchemically changed to phenylalanine or alanine (Figures 3E and 3F). Phenylalanine also stabilized the two waters in the binding pocket, resulting in a high unbinding free energy. Due to its small size, alanine, however, was unable to stabilize the water, resulting in lower binding free energy. To validate these calculations, we tested the impact of signal variants at the −1 position on both retrieval of the endogenous KDELR from the Golgi to the ER and ER retention of a reporter protein carrying the signal variant in a cellular assay. To measure the role of the −1 position on retrieval of the endogenous KDELR to the ER, cells were transfected with mScarlet-reporters carrying −1 alanine (H/KDEA) or phenylalanine (H/KDEF) variants of either the HDEL or KDEL retrieval signals (Figure 4A). Western blotting of cell extracts and extracellular medium showed that mScarlet-reporters with −1 alanine H/KDEA signals are retained less efficiently than the canonical HDEL or KDEL sequences (Figures 4B and 4C). The −1 phenylalanine KDEF and HDEF variants were retained more efficiently than the alanine variants but less efficiently than when leucine is present (Figures 4B and 4C), consistent with our PMF calculations (Figures 3E and 3F). Finally, the effect of −1 variant signals on KDELR retrieval of endogenous ER chaperones was also tested. If the exogenous signal variant had a leucine at the −1 position, luminal ER chaperones containing the KDEL (BiP and PDI) and RDEL (ERP44) retrieval signal were secreted, most likely due to competition for the receptor. This effect was reduced for HDEF or KDEF signals and lost entirely for HDEA or KDEA signals (Figures 4B and 4D). These results demonstrate the mechanistic link between the protonation state of the receptor via His12 and the importance of the water network at the base of the binding pocket for efficient retention of K/HDEL-containing proteins by the KDELR.

### Water plays a key role in stabilizing peptide binding in the KDELR

**Figure 5:**
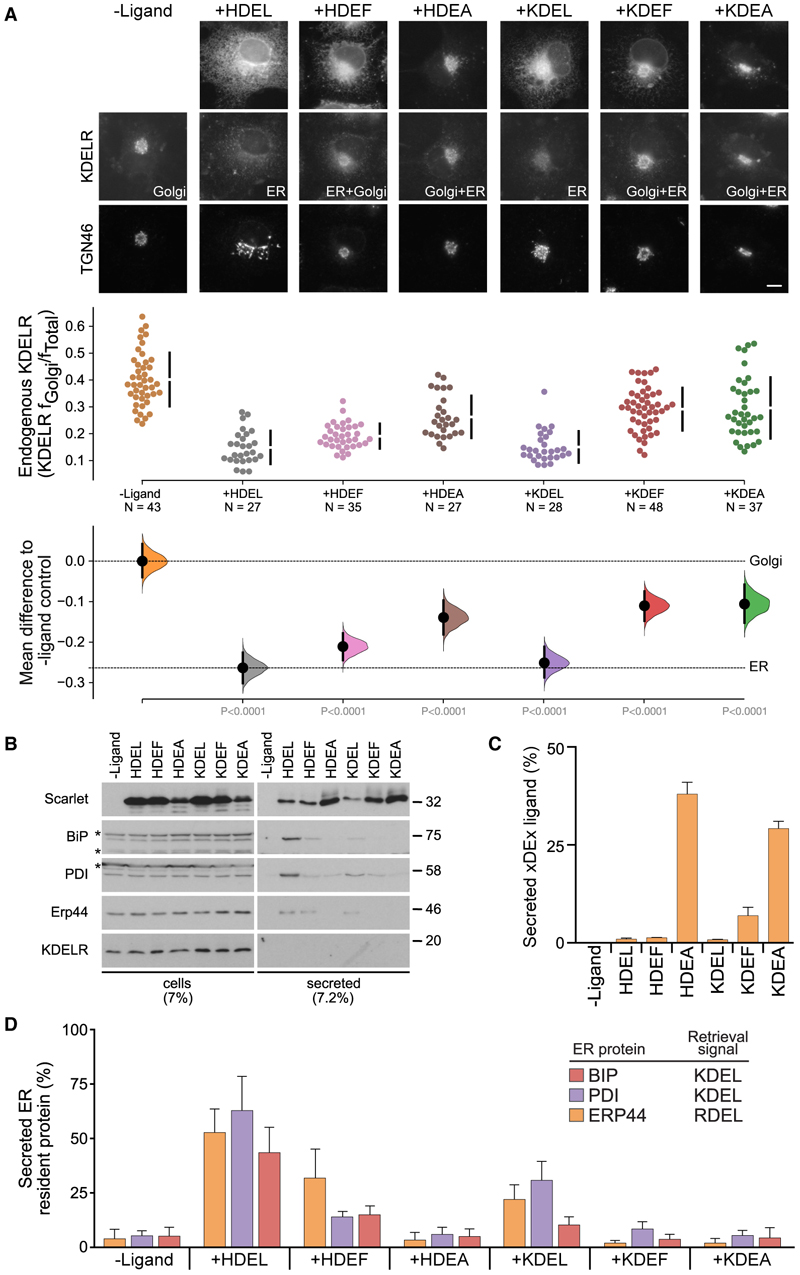
Free energy cycle of the effect of water on the binding site (A) The solvation free energy (ΔG_Solv_) is computed as the free energy of replacing the discrete waters with a continuum bulk solvent. The difference in free energy is computed as the difference in the solvation free energy between the apo and bound state. (B) The two waters in the binding pocket form a hydrogen bond network between the protein and the ligand. The space of the binding cavity (green surface) is only slightly larger than the volume of the ligand (salmon surface), which places a strong spatial constraint on the two waters. (C) The difference in solvation free energy is more positive for histidine when the binding is pH dependent (KDEL/HDEL/HDEF) but is similar for KDEA. (D) The hydrogen bond between the two waters is weaker (using occupancy as a proxy) in HDEF compared with the HDEL. (E) The terminal leucine (HDEL) exhibits a larger RMSF compared with a terminal phenylalanine (HDEF). Error bars for (C)–(E) are +/− the standard deviation (n = 3).

PMF calculations, although useful to estimate the free energy of unbinding when the protonation state of the key histidine or the terminal residue of the ligand changes, do not isolate the effect of the bound water molecules on the binding free energy, as either the terminal residue or the protonation state of the histidine could directly affect the binding. To isolate the effect of the water, we once again turned to GCMC calculations to compute the free energy of solvating the water in the binding pocket. We constructed a free energy cycle (Figure 5A) to compute the difference (ΔΔ*G*) in the solvation free energy of the water between the bound state $\left(\Delta G_{Solv}^{Bound}\right)$ and the apo state $\left(\Delta G_{Solv}^{Apo}\right)$. A negative DD*G* indicates the water is more stable in the bound state compared with the apo state. Conversely, a positive ΔΔ*G* would suggest that the water is more stable in the apo state compared with the bound state. The solvation free energy is usually defined as the free energy of removing the water and leaving a vacuum. This definition is problematic in this situation as the apo protein could host three water molecules in the binding pocket (Figures 5A and 5B). However, the same space could only host two water molecules in the bound state due to the excluded space provided by the ligand. Thus, the solvation free energy of the extra water would dominate the difference (ΔΔ*G*) between the apo and bound states. To circumvent this problem, the solvation free energy is defined as the free energy of moving the water from the binding pocket to the bulk solvent (see STAR Methods), which ensures that the solvation free energy is independent of the number of waters.

Previously, we showed that the HDEL ERS exhibited pH-dependent binding to the receptor, with the highest affinity observed under acidic conditions.^11^ Here, we observed that the water network can stabilize the bound state of the receptor only in the presence of the protonated histidine (HIP) compared with the deprotonated state (HIE) for all peptides except KDEA, where the protonation state of His12 makes very little difference to the solvation free energy of water network (Figure 5C, Table 2). It is worth noting that the ΔΔ*G* for HDEF in the His12 protonated state (HIP) was higher than the HDEL or KDEL and is close to zero, which suggests that the water network has little effect on the stability of the HDEF-bound state.

Given that the stability of the hydrogen bonding network largely determines the solvation free energy, we attempted to investigate if the phenylalanine variant at the −1 position would disrupt the water network. The water network in the binding pocket consists of three parts: the histidine (His12), which hydrogen bonds to the first water (W1), which in turn hydrogen bonds with the second water (W2), which hydrogen bonds with the C terminus of the ligand (Figures 1B and 4B). Though the hydrogen bond between the waters and the His12 or the C terminus is equally strong for HDEL and HDEF ERS sequences, the hydrogen bond between the two waters is much less stable in HDEF compared with HDEL (Figure 5D).

To understand the source of the instability of the water network, we determined the crystal structure of the receptor bound to the TAEHDEF peptide at 2.28 Å (Figure S2 and Table 1) (PDB: 7OXE). The structure was determined at pH 6.0 and is very similar to the TAEHDEL complex (PDB: 6Y7V), with a root-mean-square deviation of 0.118 Å over 118 C_α_ atoms. The W1 and W2 water molecules are observed in the binding site, consistent with our previous HDEL structure and computational analysis. In both structures, the space available for the two waters is very tight (Figures 5B and S2). Thus, a slight perturbation of the protein might compress the space and disrupt the inter-water hydrogen bond network. A leucine at the −1 position of the ERS, however, would have more flexibility to maintain the water network compared to a terminal phenylalanine, which, being more rigid, would not provide sufficient flexibility and thus result in a less stable water network. Supporting this hypothesis, the temperature factors, or B factors, of the terminal leucine in the HDEL structure are 51.3 vs. 38.2 Å^2^ for the HDEF structure, demonstrating that phenylalanine is held more rigidly. Additionally, the affinity for TAEHDEF is lower than for TAEHDEL (23 μM vs. 18 μM, Figure S3), consistent with these observations (Figure 5D). The increased flexibility of the leucine is also reflected by the higher root mean squared flexibility compared with the phenylalanine (Figure 5E). In summary, it seems likely that the increased affinity observed for the TAEHDEL peptide over the TAEHDEF peptide is due to the increased flexibility of the leucine side chain that enables more optimal coordination of the water molecules with the carboxy-terminus of the peptide within the receptor and explains the reduced ability of the HDEF ERS to retain proteins in the cell-based retrieval assay (Figure 4).

### QM/MM calculations reveal Asp9 may also be protonated

**Figure 6:**
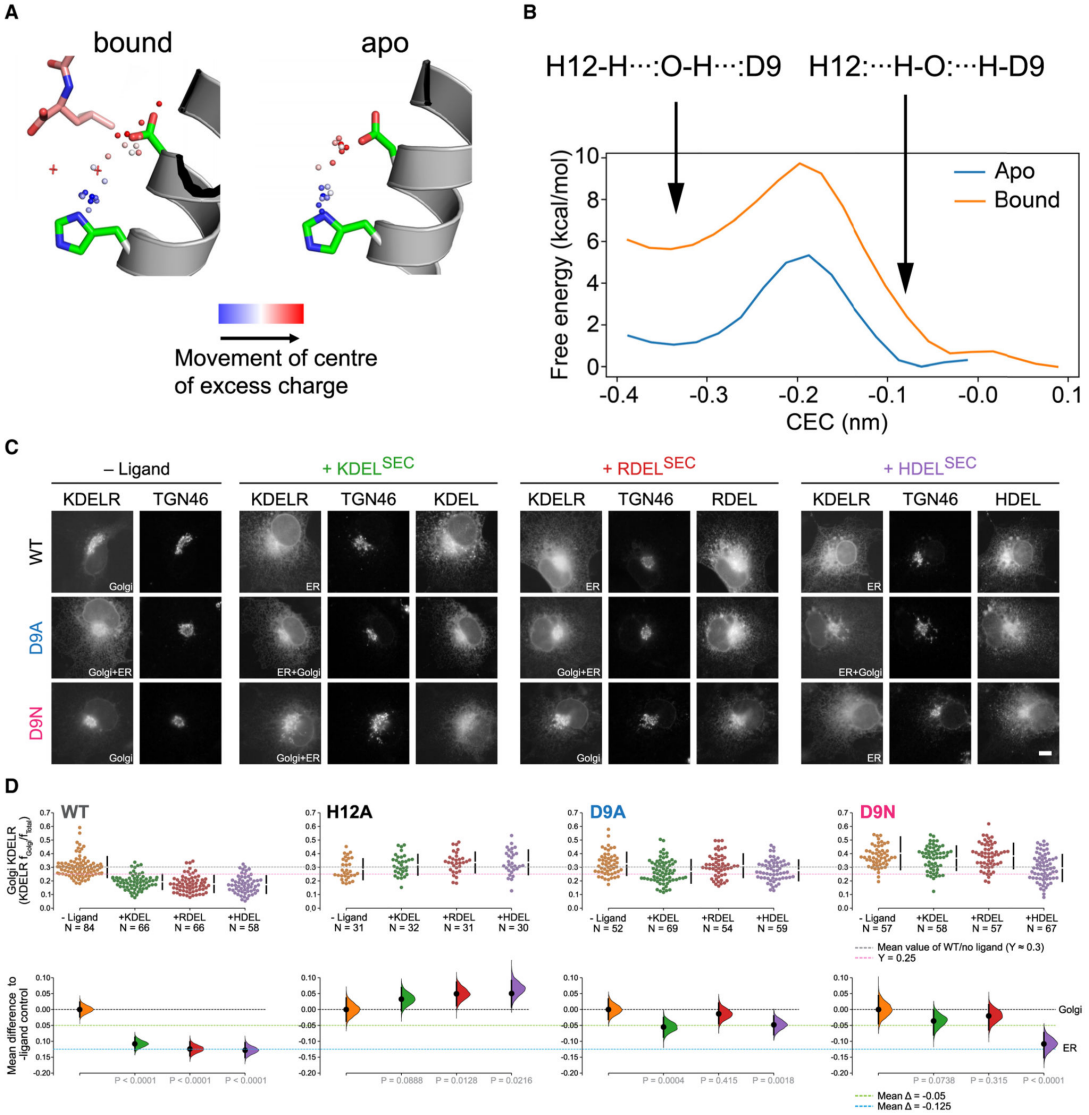
Effect of the water on proton transfer between Asp9 and His12 (A) In the bound state, a proton could be transferred from the histidine (His12/H12) to the aspartate (Asp9/D9) through the crystallographic water. A similar proton transfer can also be made in the apo state. (B) In the apo and bound state, the free energy barrier for the proton transfer (the center of excess charge) from the histidine to aspartate is similar. A larger energy barrier is observed for the proton transfer from aspartate back to the histidine in the bound state. (C) WT, D9A and D9N-mutant KDEL receptors redistribution in COS-7 cells in the absence (-ligand) or presence of K/R/HDEL (mScarlet-xDEL^sec^) ligand. TGN46 was used as a Golgi marker. The scale bar is 10 μm. (D) The fraction of wild-type and H12A, D9A, and D9N-mutant KDEL receptors localized to the Golgi was measured before (no ligand) and after challenge with different retrieval signals (K/R/HDEL) and is shown as Cummings estimation plots. Effect sizes are shown as the mean difference for K/R/HDEL comparisons against the shared ligand control. On the lower axes, mean differences are plotted as bootstrap sampling distributions. Each mean difference is depicted as a dot. Each 95% confidence interval is indicated by the ends of the vertical error bars.

Thus far, we have explored the relationship between the −1 position of the ERS, cargo retrieval efficiency, the stability of water in the binding pocket, and His12 protonation. We next wanted to explore what role the water molecules might play in the dynamics of protonation/deprotonation. In the bound state, an aspartate residue (Asp9) lies close to His12 and is linked via hydrogen bonds with one of the key waters (Figures 1 and 5A). Thus, our working hypothesis is that within the ER, the deprotonation of His12 might occur via proton transfer to Asp9 via the bridging water before finally exiting to the bulk solution. To test this hypothesis, we used umbrella sampling within a QM/MM framework to compute the free energy profile of moving the proton from the histidine to the aspartate via the bridging water for both bound and apo state (Figure 6A and STAR Methods).

The calculations reveal that the proton transition from His12 to Asp9 faces a similar barrier in the bound and apo states, but the reverse transition from Asp9 to His12 would face a much larger energy barrier in the bound state compared with the apo state (Figure 6B). This shows that the state of the deprotonated histidine and protonated aspartate is stabilized in the bound state. At first glance, this might appear counterintuitive. However, in the bound state, Asp9 sits close to the hydrophobic side chain of the terminal leucine (Figure 6A), which would favor the neutral, protonated aspartate compared to deprotonated, charged aspartate. Thus, the presence of the ERS peptide stabilizes the protonation of Asp9, severely reducing the probability of proton loss from His12. Indeed, the importance of Asp9 in ERS recognition is evident in the cell retrieval assay (Figures 6C and 6D), which shows that the Asp9Ala variant is markedly less efficient than the WT receptor for K/R/HDEL retrieval sequences.

Interestingly, while the Asp9Ala variant is severely impacted for all variations of the ERS, the Asp9Asn variant, which mimics the protonated, neutral state of the aspartate side chain, is still able to retrieve HDEL-containing cargo at ~ 80% WT levels. The retention of activity in only the asparagine variant suggests that the protonation of Asp9 plays an important role in stabilizing the ERS peptide in the binding site. The recognition of only the HDEL-containing cargo in the cell retrieval assays is consistent with the 10-fold higher affinity between HDEL peptides (Kd 0.24 μM) compared to KDEL (Kd 1.94 μM) or RDEL (RDEL Kd 2.71 μM) variants,^11^ which likely reduces the contribution of this side chain to the HDEL peptide. Taken together with the results from the QM/MM calculations, a role emerges for Asp9 in stabilizing the protonation state of His12 in the bound state, facilitating the positioning of the terminal leucine of the ERS and assisting in the correct positioning of the waters in the peptide in the binding pocket.

## Discussion

The subtle pH gradient from ER to Golgi has long been implicated in controlling protein trafficking between these two organelles.^26,27^ Recent studies on the KDELR revealed a key role for protonation of His12 in stabilizing the receptor-cargo complex through the formation of a short hydrogen bond within the receptor,^19^ which kinetically traps the receptor-cargo complex until deprotonation in the ER. Further investigation reveals this likely facilitates the protonation of His12.^22^ However, the detailed mechanism linking receptor protonation to cargo binding and release remained obscure, hampering efforts to understand how the KDELR maintains fast kinetic in the ER yet bind chaperones with high affinity in the Golgi. The crystal structures of the KDELR in complex with the TAEHDEL peptide at both neutral (representative of the ER) and acidic (representative of the Golgi) states, now provide a clear explanation for this characteristic of the KDELR trafficking system.

At neutral pH (7.0), a single water makes a bridge between the bound ERS peptide and the receptor, whereas, at acidic pH (6.0), two bridging water molecules are observed forming a stable hydrogen bond network between the carboxyl group of the ERS peptide and Tyr158 on TM6 (Figure 1B). Our GCMC calculations demonstrate this water network is only stabilized when His12 is protonated and that both the C-terminal carboxyl group and terminal leucine in the ERS also contribute to this water network (Figure 5). QM/MM calculations also suggest that the proton could sit at the Asp9 residue (indeed in the bound state it appears to be the preferred location). This would still enable the two water molecules to form a stable interaction network (see Figure 5B); thus, it may well be the case that the actual location of the proton is not that critical and moves between His12 and Asp9 during trafficking. However, to investigate this comprehensively is beyond the scope of this work.

**Figure 7:**
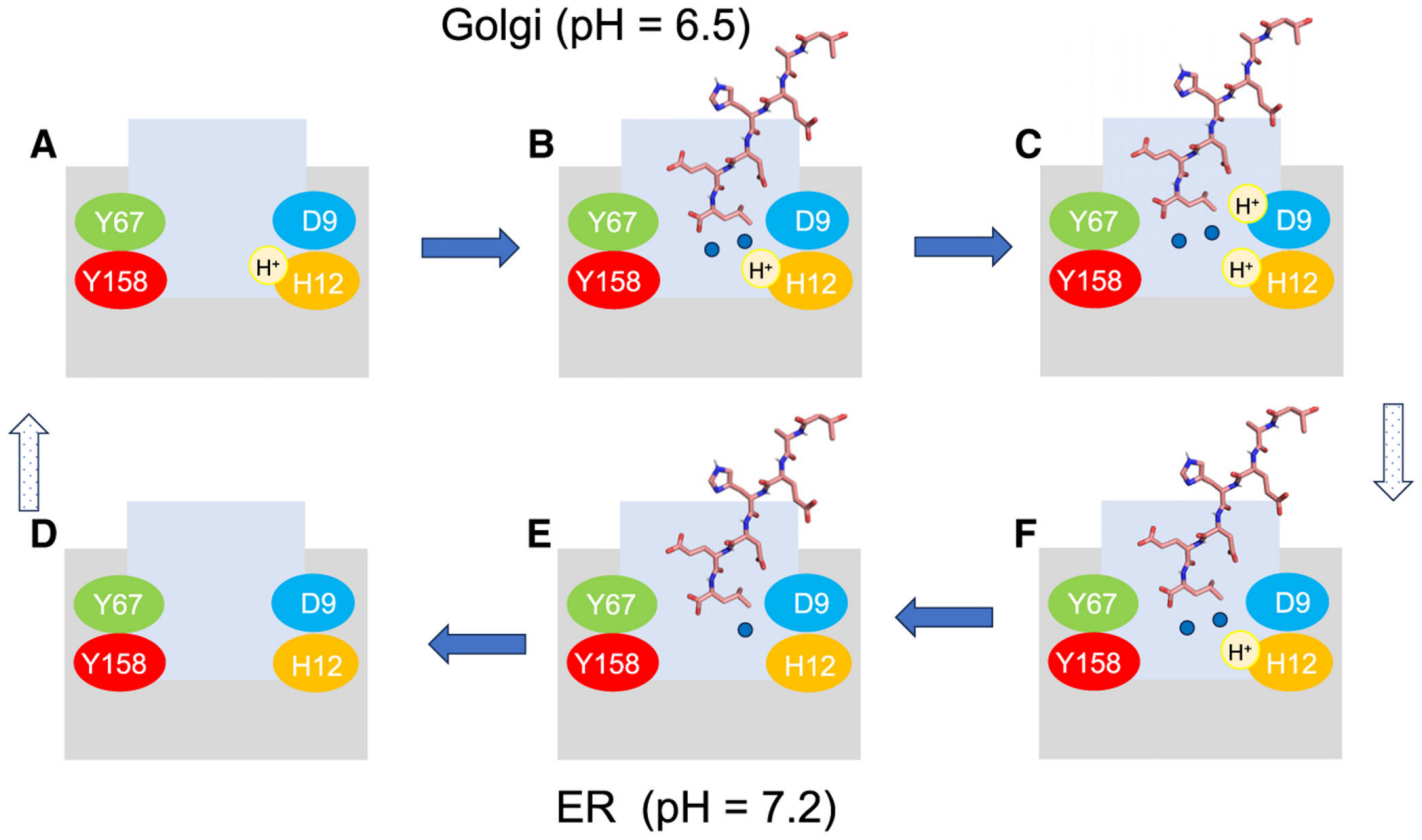
Schematic of how the different pH in the Golgi and the ER controls cargo binding The lower pH in the Golgi will favor His 12 being protonated. The proton can come from bulk or via Asp9 (A). Signal peptide binding is then stabilized by two water molecules (blue circles) that hydrogen bond to His12 (B). In addition, the proximity of Asp9 to the terminal leucine favors the protonation of the aspartate, which likely also traps the His12 in the protonated state (C). Upon arrival at the ER with a higher pH, the Asp9 will deprotonate (D), allowing the His12 to deprotonate (E). Deprotonation will destabilize the water network, and the signal peptide will unbind (F).

The relationship between the ERS peptide, water network, and protonation of the receptor is consistent with the view that within the Golgi, the receptor should have a higher affinity for the signal peptide to facilitate the recruitment of K/H/RDEL cargo proteins. However, in the ER, the affinity for the ERS peptide should drop rapidly to allow fast release of the cargo back to the ER lumen.^11,28^ To maintain fast on-rates for ERS binding in the Golgi, the KDELR must be primed and ready for peptide binding. Our results suggest the mechanism for achieving this behavior comes from the proximity of Asp9 to His12 (Figure 1), with Asp9 controlling the ability of this side chain to deprotonate (Figure 2). Taken together, our data enable us to propose a more complete mechanism of K/H/RDEL peptide recognition and how this relates to sub-cellular localization (Figure 7). In the more acidic environment of the Golgi, the receptor will be protonated on His12, and the peptide binding site will be solvated (Figure 7A). Following ERS capture, the peptide will trap the two water molecules observed in the crystal structure at the base of the binding pocket, facilitated by the hydrophobic property of leucine side chain at the −1 position and protonation of His12 of δN nitrogen (Figure 7B). Our PMF and GCMC calculations reveal the two water molecules form an important part of the binding site and play an important role in contributing to the high affinity of the receptor for K/H/RDEL peptides. The carboxyl terminus of the ERS peptide also perturbs the pKa of Asp9 to a higher value, resulting in this side chain being protonated even in the acidic environment of the Golgi (Figure 7C). Activation of the COPI retrieval signal occurs following the movement of Arg159 on TM6 to fully engage the carboxyl group of the peptide,^19^ which is stabilized by the SHB formed between Tyr158 and Asp127.^29^ This keeps the ERS peptide stably bound to the receptor while in the Golgi and activates the recruitment of COPI via release of the lysine retrieval motif on TM7. Upon transfer to the ER, deprotonation occurs via a relay system between His12 and Asp9, with Asp9 acting as the sensor for luminal pH changes (Figure 7D). Deprotonation of His12 results in destabilization of the water network, which in turn causes weakening of the interaction network between the receptor and the carboxy-terminus of the ERS peptide (Figure 7E). The large energy barrier observed in the GCMC calculations for proton transfer from Asp9 back to the His12 means that once the His12 is deprotonated, it is unlikely to be protonated again while the ERS peptide is still bound. This one-way deprotonation event thus ensures sufficient time for the water network to be destabilized, the SHB to break, and the receptor to relax back to the inactive state and release the cargo into the ER lumen. The apo protein is then trafficked back to the Golgi via COPII-coated vesicles in preparation for the next trafficking cycle (Figure 7F).

An essential feature of the KDEL trafficking mechanism is the ability to couple ERS binding with protonation of the receptor and display fast kinetics for cargo capture and release.^11^ Our data suggest that water adds an additional entropic component to the free energy of binding and release. High-affinity binding between the KDELR and cargo comes from the ordering of not just the peptide and the receptor but also two water molecules. A similar role for water has been observed in other structurally unrelated peptide-binding proteins.^30^ The water molecules in the KDELR are only stabilized in the protonated state of the receptor and following peptide binding. Thus, when the receptor deprotonates in the ER, destabilization of the water molecules will likely contribute to both the favorable thermodynamic driving force for disassembly and positively impact the kinetic release of the peptide following deprotonation.

Finally, an intriguing aspect of the KDELR is the structural homology this protein shares with solute carrier proteins.^31^ Solute carriers also display fast kinetics for ligand binding and release,^32^ which, as noted previously, is an essential feature of the KDELR. It is plausible to suppose that the adoption of a solute carrier (SLC) fold for cellular trafficking was, in part, due to the ability of solute carriers to respond dynamically to changes in environment pH and couple ion and ligand binding to fast conformational changes in the membrane.

## Star⋆Methods

### Key Resources Table

**Table:** 

REAGENT or RESOURCE	SOURCE	IDENTIFIER
Antibodies		
Polyclonal sheep α-TGN46	Bio-Rad	AHP500; RRID: AB_324049
Monoclonal mouse KDEL receptor monoclonal antibody (KR-10)	Enzo lifesciences	ADI-VAA-PT048; RRID: AB_10615208
Monoclonal mouse a-RFP,	Chromotek	6G6; RRID: AB_2631395
Polyclonal rabbit a-BIP	Abcam	ab21685; RRID: AB_2119834
Polyclonal rabbit a-PDI	ProteinTech	11245-1; RRID: AB_2298937
Monoclonal rabbit a-ERP44	Cell Signalling Technology	3798S; RRID: AB_1642195
Peroxidase-AffiniPure Donkey Anti-Rabbit IgG (H+L)	Jackson ImmunoResearch	711-035-152-JIR; RRID: AB_10015282
Peroxidase-AffiniPure Donkey Anti-Mouse IgG (H+L)	Jackson ImmunoResearch	711-035-152-JIR; RRID: AB_2340770
Donkey anti-Mouse IgG (H+L) Highly Cross-Adsorbed	Invitrogen	A-21202; RRID: AB_141607
Secondary Antibody, Alexa Fluor 488		
Donkey anti-Sheep IgG (H+L) Cross-Adsorbed Secondary Antibody, Alexa Fluor 647	Invitrogen	A-21448; RRID: AB_2535865
Bacterial and virus strains		
XL1-Blue Competent Cells	Agilent Technologies	200249
Biological samples		
Dulbecco’s modified Eagle’s medium	ThermoFisher Scientific	31966-047
Fetal Bovine Serum	Sigma-Aldrich	F9665
TrypLE Express Enzyme	ThermoFisher Scientific	12605036
Opti-MEM	ThermoFisher Scientific	11058021
Mirus LT1	Mirus Bio LLC	MIR 2306
16% Formaldehyde	Sigma-Aldrich	8908
Sodium periodate	Sigma-Aldrich	311448
Bovine Serum Albumin	Sigma-Aldrich	A4503
Saponin	Sigma-Aldrich	S-7900
Lysine HCl	Sigma-Aldrich	L8662
Chemicals, peptides, and recombinant proteins		
Peptide, TAEKDEL	Cambridge peptides	NA
Peptide, TAEHDEL	Cambridge peptides	NA
Peptide, TAEHDEL	Cambridge peptides	NA
Peptide, TAEHDEF	Cambridge peptides	NA
Peptide, 3H-TAEHDEL. 185 Mbq 106 Ci/mmol	Cambridge peptides	NA
Chemical, Cholesteryl hemicussinate (CHS)	Merck	T6399
Monolein	Merck	M7765
Dodecyl maltoside (DDM)	Anatrace	D310LA
Ultima Gold	Perkin elmer	6013326
Deposited data		
KDELR with TAEHDEL peptide at pH 7.0	This paper	7OYE
KDELR with TAEHDEF peptide at pH 6.0	This paper	7OXE
KDELR H12A with sybody 37	This paper	8APY
Experimental models: Cell lines		
HeLa S3	ATCC	CCL-2.2
COS7	ATCC	CRL-1651
Experimental models: Organisms/strains		
Yeast Strain Bj5460	ATCC	208285
Oligonucleotides		
GgKDELR_D9A_F_GATTGACCGGTGcTTT GTCTCATTTGG	This paper	N/A
GgKDELR_D9A_R_CCAAATGAGACAAAg CACCGGTCAATC	This paper	N/A
GgKDELR_D9N_F_CAGATTGACCGGTaa TTTGTCTCATTTGG	This paper	N/A
GgKDELR_D9N_R_CCAAATGAGACAAAtt ACCGGTCAATCTG	This paper	N/A
Recombinant DNA		
pcDNA3.1 hGHss-mScarlet-*H. sapiens* BiP639-654 K651H(HDEL^SEC^)	Bräuer et al., 2019.^19^	pFB9713
pcDNA3.1 hGHss-mScarlet-*H. sapiens* BiP_639-654_ K651H L654F (HDEF^sec^)	This paper	pFB10668
pcDNA3.1 hGHss-mScarlet-*H. sapiens* BiP_639-654_ K651H L654A (HDEA^sec^)	This paper	pFB10669
pcDNA3.1 hGHss-mScarlet-*H. sapiens* BiP639-654 L654F (KDEF^sec^)	This paper	pFB10670
pcDNA3.1 hGHss-mScarlet-*H. sapiens* BiP639-654 (KDEL^SEC^)	Bräuer et al., 2019.^19^	pFB9692
pcDNA3.1 hGHss-mScarlet-*H. sapiens* BiP639-654 L654F (KDEF^sec^)	This paper	pFB10670
pcDNA3.1 hGHss-mScarlet-*H. sapiens* BiP639-654 K654A (KDEA^sec^)	This paper	pFB10671
pcDNA3.1 hGHss-mScarlet-*H. sapiens* BiP639-654 K651R(RDEL^SEC^)	Bräuer et al., 2019.^19^	pFB9712
pEF5/FRT human KDELR1-GFP	Bräuer et al., 2019.^19^	pFB9693
pEF5/FRT human KDELR1 H12A-GFP	Bräuer et al., 2019.^19^	pFB9694
pEF5/FRT human KDELR1-GFP D9A	This paper	pFB10666
pEF5/FRT human KDELR1-GFP D9N	This paper	pFB10667
pDDGFP-Leu2d-GgKDELR2	Addgene	123618
pDDGFP-Leu2d-GgKDELR2_H12A	Bräuer et al., 2019.^19^	NA
pDDGFP-Leu2d-GgKDELR2_D9A	This Paper	NA
pDDGFP-Leu2d-GgKDELR2_D9N	This Paper	NA
pBXPC3H-Syb37	Addgene	123627
Software and algorithms		
Metamorph 7.5	Molecular Dynamics Inc	www.moleculardevices.com
Fiji 2.9.0/1.53t	NIH Image	http://fiji.sc/
Prism 10.0.3	GraphPad Software	www.graphpad.com
Affinity Designer 2.3.1	Serif Ltd	https://affinity.serif.com
Estimation plots	Ho et al., 2019.^36^	https://www.estimationstats.com/
ProtoMS 3.4	Woods et al., 2018.^37^	
MDAnalysis	Michaud-Agrawal et al., 2011.^38^	https://www.mdanalysis.org
Gromacs	Abraham et al., 2015^39^	https://www.gromacs.org
WHAM	Grossfield, A.^42^	http://membrane.urmc.rochester.edu/wordpress/?page_id=126
Cp2k 8.2	Kühne et al., 2020.^43^	https://www.cp2k.org
Phaser-2.8	Phenix	https://www.phaser.cimr.cam.ac.uk/index.php/Phaser_Crystallographic_Software
Buster-2.10.3	Global Phasing	https://www.globalphasing.com/buster/

### Resource Availability

#### Lead contact

Further information and requests for resources and reagents should be directed to the lead contact, Philip Biggin (Philip.biggin@bioch.ox.ac.uk).

#### Materials availability

Recombinant DNA detailed in this manuscript are freely available for academic use upon request to the corresponding authors. Simulation snap shots and skipped trajectory files can be requested from the lead contact, Philip Biggin.

### Experimental Model and Study Participant Details

The genes encoding for *Gallus gallus* (*Gg*) KDELR2 (Uniprot: Q5ZKX9) were codon optimised and cloned into a modified form of a C-terminal GFP^His^ fusion vector for expression in *Saccharomyces cerevisiae* strain BY5460. All cell lines (COS-7 and HeLa S3) are available from ATCC and were cultured as described in the STAR Methods section. COS7 were used for retrieval assay and HeLa S3 for ER secretion assays. *E. coli* XL1 blue Competent Cells were used for cloning and plasmid preparation.

### Method Details

#### KDELR crystallography and structure determination

The KDEL receptor and the His12Ala variants were expressed and purified as described previously.^19^ For the WT structure at pH 7.0, protein at 14.5 mg/ml and incubated with 6.4 mM TAEHDEL peptide for one hour on ice. Protein-laden mesophase was obtained by monoolein with protein in a 60:40 (w:w) ratio using a coupled syringe device. Crystals were set up at 20°C using precipitant 30 % PEG500DME, with 100 mM potassium thiocyanate and 100 mM HEPES pH 7.0. The WT structure with HDEF peptide bound was obtained as described previously for the HDEL peptide. For the His12Ala, the sybody receptor complex was formed as previously reported^19^ and concentrated to 21 mg/ml. Crystals were obtained in 30 % PEG400 with 100 mM HEPES pH 8.0. Phases were determined via molecular replacement using Phaser and employing PDB:6Y7V as the search model for the peptide-bound complexes and 6I6J for the sybody complex; the subsequent models were refined using BUSTER.

#### Peptide binding assays

Binding assays were performed as detailed in Bräuer, P. et al.^19^ WT or mutant protein (5 μL) in 20 mM MES pH 5.4, 40 mM Sodium Chloride, 0.01% DDM 0.0005% CHS was incubated with 5 μL of 3H-TAEHDEL (Cambridge Research Biochemicals, UK) at 20 nM at 20°C for 10 min. Using a vacuum manifold, the reaction was then filtered through a 0.22 μm mixed cellulose ester filters (Millipore, USA). Filters were washed with 2 x 0.5 mL cold buffer. The amount of peptide remaining bound was measured using scintillation counting in Ultima Gold (Perkin Elmer). Experiments were performed at least three times, independently, to generate an overall mean and standard deviation (s.d).

#### Retrieval assays

Retrieval assays were performed as described in Gerondopoulos et al.^11^ Briefly, COS-7 cells were grown on coverslips and transfected with KDELR-GFP and/or mScarlet-ligand (+xDEx ligand) or KDELR-GFP and pcDNA3.1 (− ligand) with TransIT LT1. After a further 18 hr, cells were fixed for 2 hours and subsequently permeabilised with saponin for 30 min. Primary and secondary antibody staining was performed sequentially for 60 min in permeabilisation solution at 22°C. Coverslips were mounted in Mowiol and imaged on an Olympus BX61 upright microscope. The signal for the KDEL receptor (integrated pixel intensity) was measured in individual cells using FIJI^33^ for the Golgi region defined by the TGN46 Golgi marker and for the entire cell in the presence (+) and absence (-) of ligand. The fraction of KDEL receptor in the Golgi apparatus was calculated by dividing the Golgi signal by the total cell signal. To estimate the effect sizes and significance of receptor mutations for ligand-mediated ER retrieval pooled data was analysed in R using the open-source package DABEST.^34–36^

#### ER secretion assays

ER chaperone secretion assays were performed as described in Gerondopoulos et al.^11^ Briefly HeLa S3 cells were transfected with mScarlet-ligand (+xDEL ligand) or pcDNA3.1 (− ligand), and allowed to express the respective proteins for 24 hr. The media were TCA precipitated and both cell and media were resuspended and boiled in SDS-PAGE sample buffer. All samples were analysed by Western blotting and signals on films were measured by densitometry in FIJI.^33^ Data were plotted as bar graphs in GraphPad Prism.

#### Grand canonical Monte Carlo (GCMC) simulations

The GCMC calculations were performed as described by us previously^22^ with ProtoMS 3.4.^37^ The GCMC calculations were performed for eight setups, which are the permutation of four ligands, HDEL, KDEL, HDEF, and KDEA, with two protonation states of the histidine (HIE/HIP). The GCMC box has a dimension of 3.23 * 3.38 * 5.78 Å and is centred at the centre of the two waters in the water network. Five repeats were run with Adams value ranging from -9.0 to -24.0 with a step of 0.5, where a production run of 200000000 steps was preceded by an equilibration run of 20000000 steps. The analysis of the trajectory was performed with MDAnalysis.^38^

#### Potential of mean force calculations

Umbrella sampling was performed to compute the free energy of removing the HDEX ligand from the binding pocket with Gromacs 2020.^39^ The KDELR protein was embedded in the DLPC lipid^40^ solvated with TIP3P water^41^ and ionised to a NaCl concentration of 0.15 M. The setup of the protein-lipid system was described by us previously.^11^ Four different setups were prepared: protonated H12 with HDEL ligand, deprotonated H12 with HDEL ligand, protonated H12 with HDEF ligand and protonated H12 with HDEA ligand. The collective variable was defined as the distance between the binding pocket, which is defined as the centre of the mass of Cα atoms of residue 9, 44, 64, 124, and 162, to the N-terminus of the HDEX peptide (N atom). Three repeats were run for each case with umbrella sampling windows ranging from 1.8 nm to 3.3 nm with a step of 0.1 nm. The production run was performed for 200 ns with an equilibrium run of 200 ps and replica exchange with an interval of 1000 steps. The results were analysed with WHAM^42^ and the error was the standard deviation of the estimate.

#### QM/MM calculations

The Apo and HDEL-bound system were represented with amber topology and the MD engine is cp2k 8.2.^43^ The H12, D9, E127, Y158 and the bridging water were treated at the QM level using the BLYP-D3 functional.^44^ The equilibrium run was performed with DZVP-MOLOPT-SR-GTH basis set and the production run was performed with the TZV2P-MOLOPT-GTH basis set.^45^ The equilibrium run was performed for 100 fs and the production run was performed for 20 ps with a time step of 0.5 fs. The CSVR thermostat^46^ was used with a time constant of 50 fs to restrain the temperature to 310 K. The umbrella sampling was performed with plumed.^47^ The collective variable was defined as

$$CEC=D_{H\text{12:}N-H\text{12:}H}-D_{W\text{:}0-H\text{12:}H}+D_{W\text{:}0-W\text{:}H1}-D_{D9\text{:}0-W\text{:}H1}+D_{W\text{:}0-W\text{:}H2}-D_{D9\text{:}0-W\text{:}H2}$$

Where *H*12 : *N* and *H*12 : *H* are the Nδ of the histidine and the corresponding proton. The *W* : 0 and *W* : *H*1, *W* : *H*2 are the oxygen and the two protons of the bridging water. The *D*9 : 0 is the oxygen in the Asp9 side chain. The *D* stands for the distance between the two atoms.

For the apo protein, 17 windows from 0 to -0.4 with a step of 0.025 nm were constructed. For the bound protein, 21 windows from 0.1 to -0.4 with a step of 0.025 nm were constructed. The force constant of the collective variable was set to 20000 kj/mol/nm^2^. The result was analysed with WHAM.^42^

### Quantification and Statistical Analysis

Means and standard error of the mean for the peptide binding, retrieval and secretion assays were calculated using R.^35^ Mean and standard deviations for computationally derived data were computed with python scripts that employed scipy.^48^ Statistical details can be found in the relevant figure legends. The data was assumed to adopt a normal distribution. X-ray crystallographic data collection and refinement statistics are summarized in Table 1.”

## Supplementary Material

Supplemental information can be found online at https://doi.org/10.1016/j.str.2024.03.013.

Document S1. Figures S1–S3.

## Figures and Tables

**Table 1:** Summary of crystallographic data collection and statistics

	KDELR2 – TAEHDEL pH 7.0	KDELR2 – TAEHDEF pH 6.0	KDELR2 – Syb37 H12A
Data collection			
PDB	7OYE	7OXE	8APY
Space group	P 2_1_	P 2_1_	P 2_1_ 2_1_ 2_1_
Cell dimensions a, b, c (Å)	48.02, 38.03, 62.51	47.38, 37.53, 62.68	45.19, 71.09, 133.03
Cell angles α, β, γ (°)	90, 95.42, 90	90, 95.22, 90	90, 90, 90
Wavelength (Å)	0.9698	0.9999	0.9686
Resolution (Å)	38.03−2.62 (2.69−2.62)	62.28−2.28 (2.35−2.28)	42.79−2.34 (2.40−2.34)
R_pim_^a^	9.8 (120)	15.9 (77.4)	9.4 (121.8)
I/σI^a^	5.6 (1.0)	5.1 (0.9)	9.4 (1.0)
CC1/2^a^	98.7 (53.1)	98.5 (30.0)	99.7 (40.6)
Completeness (%)^a^	98.4 (98.9)	99.3 (100)	99.6 (99.9)
Multiplicity^a^	3.1 (3.1)	3.2 (3.2)	6.2 (6.0)
Refinement			
Resolution (Å)	36.02−2.62	39.41−2.28	42.79−2.34
Number of reflections	6853	10122	18676
R _work_/R _free_	23.3/27.5	24.4/28.7	26.81/32.87
Average B, all atoms (Å^2^)	66.2	39.63	67.88
Rms deviations			
Bond lengths (Å)	0.009	0.009	0.007
Bong angles (°)	0.88	0.85	1.13
Ramachandran statistics Favored/outliers (%)	98.58/0.0	99.06/0.0	97.51/0.31
Molprobity score	1.42	1.20	2.23

a Highest resolution shell is shown in parenthesis.

**Table 2:** ΔΔG of the contribution from discrete solvent on binding (kcal/mol)

Peptide ligand	Protonation State		
	HID	HIE	HIP
KDEL	1.2 ± 0.3	9.2 ± 0.4	−4.2 ±0.4
HDEL	0.6 ± 0.2	8.3 ± 0.3	−3.8 ± 0.4
HDEF	4.6 ± 0.3	9.6 ± 0.4	0.3 ± 0.4
KDEA	2.0 ± 0.3	2.0 ± 0.6	1.2 ± 0.4

## Data Availability

Atomic coordinates for the models have been deposited in the Protein Data Bank (PDB) under accession codes 7OYE, 7OXE and 8APY. Data generated or analysed during this study are included in the manuscript and supporting files. Any additional information required to re-analyze the data in this paper is available from the lead contact upon request. This paper does not report original code.

## In brief

Wu et al. use a combination of X-ray crystal structures of the KDEL receptor at neutral and acidic pH with advanced computational methods and cell-based assays to explain at the atomic level how the cell performs pH-dependent retrieval of cargo proteins from the Golgi to the ER.

## Highlights

- KDELR X-ray structures at neutral and acidic pH reveal distinct bridging waters
- At neutral pH, only one water is observed, but at acidic pH two waters are seen
- GCMC calculations show the two-water network is stabilized by a protonated histidine
- The data reveal the mechanism of pH sensing in a trafficking receptor
