# Supplementary material for: Molecular basis for pH sensing in the KDEL trafficking receptor
Source: Structure. Author manuscript; Available in PMC 2025 Jul 8. (PMC7617889; doi:10.1016/j.str.2024.03.013)
Supplement: Document S1. Figures S1–S3. [file EMS206717-supplement-Document_S1__Figures_S1_S3_.pdf]

Structure, Volume *32*

Supplemental Information

Molecular basis for pH sensing

in the KDEL trafficking receptor

Zhiyi Wu, Kathryn Smith, Andreas Gerondopoulos, Tomoaki Sobajima, Joanne L. Parker, Francis A. Barr, Simon Newstead, and Philip C. Biggin

SUPPLEMENTARY INFORMATION

Molecular basis for pH sensing in the KDEL trafficking receptor.

Zhiyi Wu[1,4,†], Kathryn Smith[2,†], Andreas Gerondopoulos[2], Tomoaki Sobajima[2], Joanne L. Parker[2,3], Francis A. Barr[2], Simon Newstead[2,3]* and Philip C. Biggin[1,2]*.

[1] Structural Bioinformatics and Computational Biochemistry, Department of Biochemistry, University of Oxford, Oxford, OX1 3QU, UK

[2] Department of Biochemistry, University of Oxford, Oxford, OX1 3QU, UK

[3] Kavli Institute for Nanoscience Discovery, University of Oxford, Oxford, OX1 3QU

[4]Present address: Exscientia, Oxford Science Park, The Schrödinger Building, Oxford OX4 4GE

[†]These authors contributed equally.

* To whom correspondence should be addressed:

Simon.newstead@bioch.ox.ac.uk or *philip.biggin@bioch.ox.ac.uk*

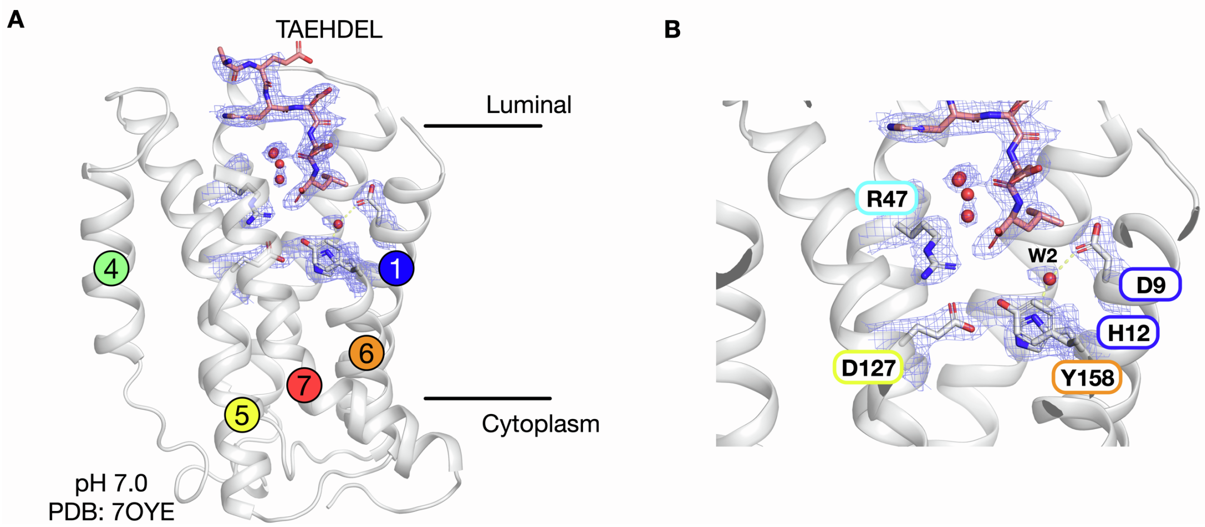

Figure S1. Partial electron density maps, related to Figure 1**.** (**A**) Crystal structure of the chicken KDELR2 receptor bound to TAEHDEL peptide at pH 7.0. Helices are labelled and coloured as in the main text Figure 1. The 2mFo-DFc election density map (blue mesh) contoured at 1σ is shown for the binding site side chains (grey sticks) and TAEKDEL peptide (salmon sticks). Hydrogen bonds (yellow dashed lines) are shown between the water coordinating His12 and Asp9. **(B)** Zoomed in view of the ERS binding site with the side chains labelled and coloured according to helix.

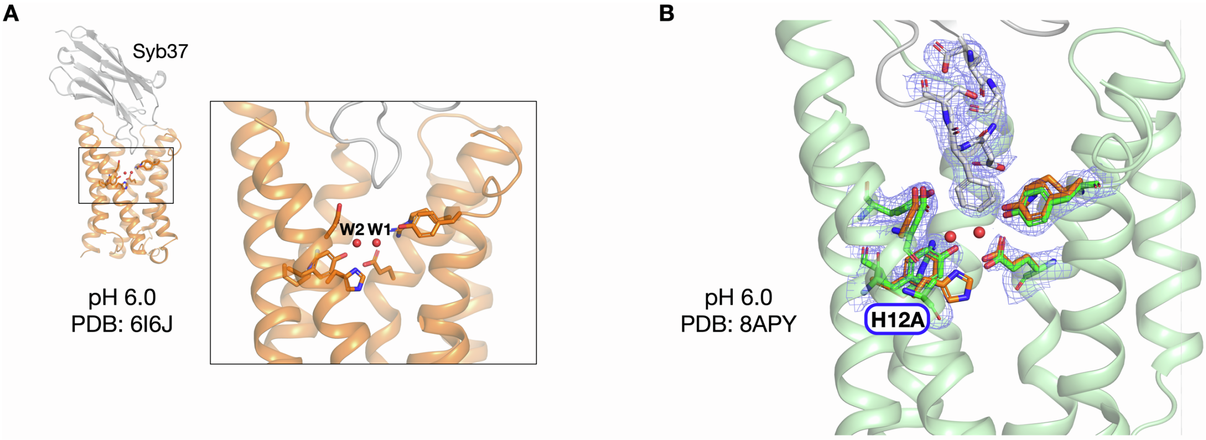

Figure S2. Additional structural data highlighting the evidence for water molecules in the binding pocket, related to Table 1 and Figure 5. (**A**) Crystal structure of the chicken KDELR2 receptor (orange) bound to synthetic nanobody (Sybody 37) (grey). A zoomed in view of the binding site is shown in the box to the right. The key side chains in the ER retrieval signal binding site are shown as sticks. The two water molecules, W1 and W2, coordinated by the ERS binding site side chains are shown (red spheres). (**B**) Crystal structure of the H12A variant of chicken KDELR2 (green) bound to sybody 37 (grey). Overlaid on the binding site are the side chains and water molecules from the WT receptor shown in (A). The 2mFo-DFc election density map (blue mesh) contoured at 1σ is shown for the binding site side chains.

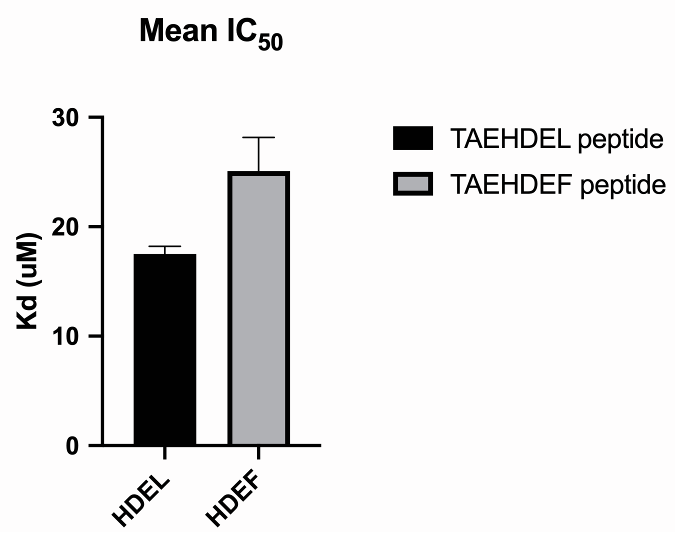

Figure S3. Affinity of different cargo signals, related to Figure 5. Mean IC_{50} values calculated for the TAEHDEF and TAEHDEL peptides competing ^{3}H-TAEKDEL peptide. Error bars are standard deviation of the mean (n = 3).
